# Quantitative, Multi-institutional Evaluation of MR Thermometry Accuracy for Deep-Pelvic MR-Hyperthermia Systems Operating in Multi-vendor MR-systems Using a New Anthropomorphic Phantom

**DOI:** 10.3390/cancers11111709

**Published:** 2019-11-02

**Authors:** Sergio Curto, Bassim Aklan, Tim Mulder, Oliver Mils, Manfred Schmidt, Ulf Lamprecht, Michael Peller, Ruediger Wessalowski, Lars H. Lindner, Rainer Fietkau, Daniel Zips, Gennaro G. Bellizzi, Netteke van Holthe, Martine Franckena, Margarethus M. Paulides, Gerard C. van Rhoon

**Affiliations:** 1 Department of Radiation Oncology, Erasmus MC Cancer Institute, 3015 GD Rotterdam, The Netherlands; h.t.mulder@erasmusmc.nl (T.M.); g.bellizzi@erasmusmc.nl (G.G.B.); tenvijver@xs4all.nl (N.v.H.); m.franckena@erasmusmc.nl (M.F.); m.m.paulides@tue.nl (M.M.P.); g.c.vanrhoon@erasmusmc.nl (G.C.v.R.); 2 Department of Internal Medicine III, Ludwig-Maximilians University Hospital, 81377 Munich, Germany; bassim.aklan@googlemail.com (B.A.); lars.lindner@med.uni-muenchen.de (L.H.L.); 3 Department of Paediatric Haematology and Oncology, Medical Faculty, Heinrich-Heine-University Düsseldorf, 40225 Düsseldorf, Germany; mils@med.uni-duesseldorf.de (O.M.); wessalowski@med.uni-duesseldorf.de (R.W.); 4 Department of Radiation Oncology, University Hospital Erlangen, 91054 Erlangen, Germany; manfred.schmidt@uk-erlangen.de (M.S.); rainer.fietkau@uk-erlangen.de (R.F.); 5 Department of Radiation Oncology, University Hospital Tübingen, 72076 Tübingen, Germany; ulf.lamprecht@med.uni-tuebingen.de (U.L.); daniel.zips@med.uni-tuebingen.de (D.Z.); 6 Department of Radiology, Ludwig-Maximilians University Hospital, 81377 Munich, Germany; 7 Center for Care and Cure Technologies Eindhoven (C3Te), Department of Electrical Engineering, Eindhoven University of Technology, 5600 MB Eindhoven, The Netherlands

**Keywords:** quality assurance, radiofrequency hyperthermia, magnetic resonance imaging-guided hyperthermia, magnetic resonance thermometry, thermistor probe, anthropomorphic phantom

## Abstract

Clinical outcome of hyperthermia depends on the achieved target temperature, therefore target conformal heating is essential. Currently, invasive temperature probe measurements are the gold standard for temperature monitoring, however, they only provide limited sparse data. In contrast, magnetic resonance thermometry (MRT) provides unique capabilities to non-invasively measure the 3D-temperature. This study investigates MRT accuracy for MR-hyperthermia hybrid systems located at five European institutions while heating a centric or eccentric target in anthropomorphic phantoms with pelvic and spine structures. Scatter plots, root mean square error (RMSE) and Bland–Altman analysis were used to quantify accuracy of MRT compared to high resistance thermistor probe measurements. For all institutions, a linear relation between MRT and thermistor probes measurements was found with *R*^2^ (mean ± standard deviation) of 0.97 ± 0.03 and 0.97 ± 0.02, respectively for centric and eccentric heating targets. The RMSE was found to be 0.52 ± 0.31 °C and 0.30 ± 0.20 °C, respectively. The Bland-Altman evaluation showed a mean difference of 0.46 ± 0.20 °C and 0.13 ± 0.08 °C, respectively. This first multi-institutional evaluation of MR-hyperthermia hybrid systems indicates comparable device performance and good agreement between MRT and thermistor probes measurements. This forms the basis to standardize treatments in multi-institution studies of MR-guided hyperthermia and to elucidate thermal dose-effect relations.

## 1. Introduction

Hyperthermia is an emerging treatment modality for malignant cancer which is performed in conjunction with chemotherapy and/or radiotherapy [1,2]. The aim of hyperthermia systems is to ensure tumour selective heating, ideally within the narrow range of 40–44 °C, while restricting the heating of surrounding healthy tissues to prevent toxicity [3]. Clinical trials have reported thermal dose-effect relations [4,5,6,7,8,9] which strongly supports the need for measuring 3D-temperature combined with precise control of the generated heating pattern to apply the maximum achievable thermal dose. The implementation of quality assurance (QA) measures is essential to assure reliable system performance and to maximize the delivery of a specific thermal dose to achieve effective therapy. We previously reported that magnetic resonance thermometry (MRT) measurements using phantoms provide 3D-QA capabilities [10] to evaluate the performance of MR-compatible hyperthermia applicators. Earlier, an elliptical cylindrical phantom containing bone structures was extensively used in the QA evaluation of the first BSD-2000-3D MR-compatible system (Pyrexar Medical Corp., Salt Lake City, UT, USA) [11]. In this work we use a human shape representative phantom including a realistic pelvic bone and spine structures. To our knowledge, such a realistic anthropomorphic phantom has not been utilized before to evaluate the accuracy of MRT against gold standard temperature probes used in clinical treatments. In addition, this is the first comprehensive analysis of MRT accuracy across multiple clinical MR-compatible systems installed in different hyperthermia institutions. 

Current clinical QA guidelines for the application of hyperthermia [12,13,14] focus on non-MR compatible heating systems, which have a limited number (usually up to eight) of intraluminal (minimally invasive) and invasive/interstitial thermometry probes [15,16]. Such thermometry using high resistance thermistor probes, fibre-optic temperature probes or thermocouples is considered the gold standard to control the clinical delivery of the hyperthermia treatment. The drawback of using probes is that they provide only temperature data with a limited spatial and temporal resolution at certain locations or along the implanted catheter, by moving the probe inside. In addition, interstitial thermometry is associated with potential risks such as haemorrhages, infections, acute side effects, and furthermore, it is considered relatively time-consuming. Therefore, it has low acceptance by patients and physicians [17,18]. 

MRT offers the unprecedented advantage of non-invasively monitoring temperature changes in the treated volume and in the surrounding regions. Clinical MR-guided hyperthermia was first introduced in the late 1990s and early 2000s [19,20,21]. Currently, the combination of an MR-compatible applicator within an MR-system, so-called MR-hyperthermia hybrid system is considered the most advanced hyperthermia technology for treatment of deep pelvic cancers and extremities providing 3D non-invasive thermometry. While installation of MR-hyperthermia hybrid systems is still limited to a few institutions worldwide, the number of systems within the hyperthermia community has increased during the last decade. The growing interest in monitoring hyperthermia treatments with full 3D-dosimetry is leading to more institutions actively developing MR-hyperthermia hybrid systems for other tumour locations as in head and neck [22], and brain [23] and the involvement of a broader scope of MR-vendors. Currently, the only commercial MR-compatible deep hyperthermia applicators in clinical use are the BSD-2000-3D with the Sigma-Eye and Universal Applicator (Pyrexar Medical Corp., Salt Lake City, UT, USA) for the treatment of pelvic and extremity tumours in adults. These applicators have been integrated into MR-systems of three of the major MR-suppliers (GE, Philips, and Siemens). Since QA procedures for these hybrid systems are minimal, there is a strong need to investigate if these devices have comparable performance in heating pattern quality and in temperature monitoring, which are the prerequisites for multi-institutional studies. 

Gellermann et al. evaluated the feasibility of various MRT methods for deep hyperthermia in a Siemens MR-scanner and using the first MR-compatible Sigma-Eye applicator in a homogeneous phantom [24]. They concluded that the evaluated MRT methods achieved a temperature resolution with respect to thermistor probe measurements in the range of ±0.5 to ±1 °C. Additionally, the same group evaluated the accuracy of the proton-resonance frequency shift (PRFS) method, which is the most widely used MRT method, versus thermistor probe measurements for their deep hyperthermia system using a heterogeneous phantom [11]. They found that the PRFS method could reproduce probe measurements with an accuracy of ±0.4 to ±0.5 °C. Following, Tarasek et al. evaluated the accuracy of the PRFS method and thermistor probes for a head and neck applicator operating within a GE system and they also obtained an overall temperature accuracy within <0.5 °C [25]. Furthermore, Gellermann et al. studied the potential of MRT in patients with recurrent rectal carcinoma with presacral location [26]. They concluded that MRT was feasible for regional hyperthermia and they found a correlation between MRT and tumour related thermistor probe readings of *R*^2^ = 0.67. In a follow-up study, they evaluated MRT for monitoring hyperthermia treatments in patients with soft tissue sarcomas of the lower extremities and pelvis [27]. They found an excellent correlation between MRT and thermistor probe readings (*R*^2^ = 0.96). In a more recent study, Craciunescu et al. [28] evaluated the accuracy of MRT vs. fibre optic probe measurements for high-grade extremity soft tissue sarcomas using a GE Signa Excite scanner and an in-house developed phased array hyperthermia applicator. They obtained excellent agreement between MRT and invasive thermometry probes with a mean difference between both techniques of 0.91 °C.

**Figure 1:**
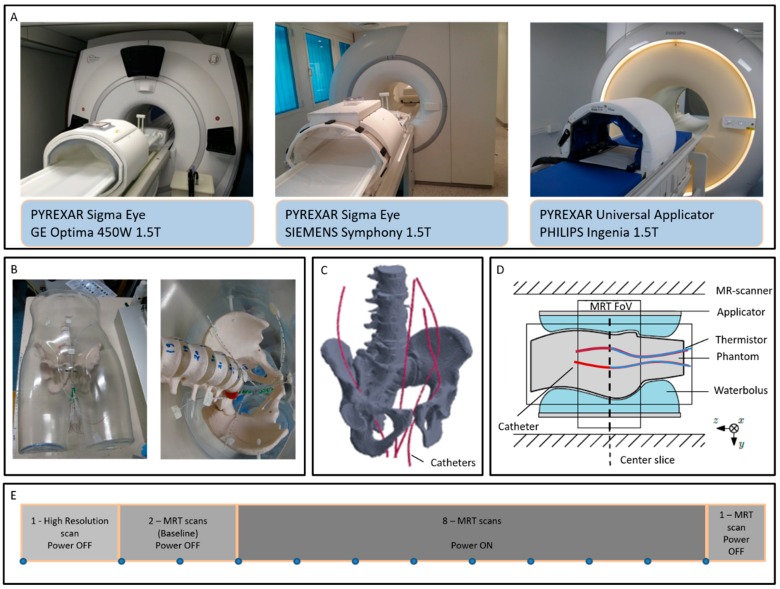
Pyrexar BSD2000-3D-MR Sigma Eye and Pyrexar BSD2000-3D-MR Universal hyperthermia applicators operating inside GE, Siemens and Philips MR-scanners (**A**), newly developed anthropomorphic phantom and close view of the catheter positioning (**B**), 3D schematic of the phantom bone structure and catheter locations (**C**), schematic cross-section of the positioning of the phantom inside the MR-scanner and monitoring of the central axial slide (**D**), and sequence of the performed MR scans (**E**).

The MR-compatible deep hyperthermia applicators installed at the five evaluated hyperthermia institutions operate within MR-systems of three major MR-vendors at a field strength of 1.5 T (see Figure 1A). Hence, the hybrid systems may experience differences in magnet configuration, quality of gradients, protocol parameters of the PRFS-method, the image quality, etc., all potentially influencing the accuracy of the MRT. To the best of our knowledge, there is no study comparing and evaluating the performance of the different hyperthermia applicators operating in the multi-vendor MR-systems with respect to MRT accuracy. Therefore, to improve comparison of clinical results from different institutions, we systematically and quantitatively assessed the temperature accuracy of the MRT against the thermistor probes, as gold standard, across the five hybrid-systems installed at the University Hospitals of Düsseldorf (Siemens, Sigma Eye), Erlangen (Siemens, Sigma Eye), Munich (Philips, Universal Applicator) and Tübingen (Siemens, Sigma Eye), in Germany and the Erasmus MC Cancer Institute (GE, Sigma Eye) in Rotterdam, The Netherlands. The long-term aim of this work is to contribute to the development of comprehensive QA-guidelines for existing and new MR-guided hyperthermia systems as a solid foundation for multi-institution clinical trials and to elucidate thermal dose-effect relations.

## 2. Methods and Materials

### 2.1. Phantom Development

Two similar, but not identical, anthropomorphic phantoms with representative shape and dimensions of an adult male were specifically developed to evaluate the MR-heating performance of the hybrid systems at the different institutions. A transparent male mannequin of wall thickness 4 mm (BP01037, Shophouse, Ede, The Netherlands) with a detailed anatomical body shape was used as the phantom shell. Artificial plastic pelvic, spine bones and discs of a full-body anatomical skeleton (180 cm) (VM101, Vosmedisch, Amsterdam, The Netherlands) were positioned inside the shell (see Figure 1B,C). While phantoms containing bone structures have been previously presented in literature for deep hyperthermia evaluations [11], this work reports for the first time the use of human shape representative phantoms. Holes with diameter of 2.5 mm spaced ~15 mm apart were drilled on the phantom shell in the perineum area. Closed-tip catheters with 350 mm length, 1.10 mm ID and 2 mm OD (Somatex, Medical Technologies GmbH, Berlin, Germany), i.e., the same catheters used clinically in deep hyperthermia treatments at the Erasmus MC Cancer Institute, were inserted through the drilled holes and positioned inside and around the bone structure. Dielectric properties of the phantom shell and artificial bones were relative permittivity of 2.8 and effective conductivity of 0 S/m at 100 MHz. The phantom shell was filled with a mixture of sodium benzoate, agar and deionized water (10 g sodium benzoate and 20 g agar per 1000 g demineralized water). Dielectric properties of the phantom were measured with a Dielectric Assessment Kit (DAK, Speag, Zurich, Switzerland) at room temperature of 21 °C. The measured values were relative permittivity of 78.6 ± 0.03 and effective conductivity of 0.41 ± 0.0002 S/m at 100 MHz.

The two phantoms were produced following the same methodology. One of the phantoms was always used to evaluate focus steering for centric heating target while the other one was always used to evaluate focus steering for eccentric heating target. Only one measurement can be performed per phantom per day due to the phantom’s need to cool down and stabilize its temperature. Therefore, two similar phantoms were produced to perform centric and eccentric measurements in one day. The centre of the phantom was defined and labelled on the phantom surface by a graphite cylinder of 12mm length and 0.8mm outer diameter that is visible in CT. Four high resistance thermistor probes (Pyrexar Medical Corp., Salt Lake City, UT, USA) were inserted in the catheters and the alignment of the temperature probes with the centre of the phantom was determined by CT-guidance. The labelled center of the phantom was then positioned at the centre of the Pyrexar BSD2000-3D-MR Sigma Eye or Pyrexar BSD2000-3D-MR Universal Applicator (Figure 1D). In order to reduce the associated spatial and temporal uncertainty, the probes were kept fixed at the central position and no mapping of the probes was performed. 

### 2.2. QA Measurements

Phantoms were acclimatised at the MR-room temperature starting the day before performing the MR-measurements in order to exclude any residual temperature gradients. The same set of measurements was performed at each of the five European hyperthermia institutions with a hybrid BSD2000-3D MR-system. All systems are maintained following the service contract from the supplier (Dr. Sennewald Medizintechnik GmbH, Munich, Germany). Focus steering was applied directly in these ‘clinical’ ready to use systems. Hence, the measurements are considered to provide real time and realistic reflection of the daily performance of the system. With the phantoms positioned in the same way, steering accuracy of the applicator focus was assessed for two steering conditions, aiming to have a centric focus at the (0, 0, 0) cm location and a focus at an eccentric location of (3, 0, 0) cm. High resistance thermistor probes and MRT measurements were performed applying equal power to each of the twelve channels of the applicator with a total clinically relevant power of 600W for a duration of approximately 12 min. 

The PRFS method was used to monitor the MRT during the measurements [10,29,30,31,32]. Standard gradient-echo (GRE) sequences with two echo times were used. A high-resolution scan was used to verify the correct positioning of the phantom, detect air pockets inside the water bolus and delineate the fat-like references attached inside the applicator which are used to compensate for B0 drift [10] during the measurements. Two MRT scans were performed as a baseline without heating. Afterwards, eight consecutive MRT scans were acquired with RF-power to the hyperthermia applicator switched on, consecutively followed by a ninth scan with RF-power switched off, as shown in Figure 1E. All scans were performed with the following parameters: 25 axial slices, slice thickness of 10 mm, no separation between slices, and a field of view (FoV) of 50 × 50 cm. Table 1 shows the main parameters of the scans at the five institutions as used in their clinical routine and the reported measurements here. 

### 2.3. Data Analysis 

MR-data was processed with the MRT package Sigma Vision Advance (Dr. Sennewald Medizintechnik GmbH, Munich, Germany) to obtain the MR-3D thermal maps per scan. The MR-3D thermal maps were then imported into Matlab (version R2016b, MathWorks, Natick, MA, USA) for quantitative analysis. In the first step, the catheter locations were identified in the magnitude MR-images. To evaluate the MRT, a region of interest (ROI) of 15.625 mm-side lengths centred in each catheter location was selected in the central axial slice. This ROI dimension corresponds with the minimum square containing 4 × 4 pixels for Institutions 2, 4, and 5 (reconstruction matrix: 128 × 128 pixels, pixel size 3.906 mm). The centre 2 × 2 pixels of the ROI containing the catheters were neglected from the calculation to prevent the inclusion of pixels containing signal void from the plastic catheters. For Institutions 1 and 3 (reconstruction matrix: 256 × 256 pixels, pixel side length of 1.953 mm), to obtain the same ROIs of 15.625 mm-side lengths, 8 × 8 pixels were evaluated, from which the centric 4 × 4 pixels containing the catheters was neglected. The mean and standard deviation values were obtained from the ROI of each time-specific thermal map. Thermistor probes measurements were taken every 10 seconds over the full heating and cooling period.

A qualitative comparison of the temperature increase was performed by including the temperature readings from the four thermistor probes and their related MRT as evaluated in the four ROI (mean and standard deviation) for each specific time-scan. For quantitative comparison, scatter plots of both temperature measurement methods with determination of regression lines and *R*^2^ were used. Regression analysis quantifies the linear relationship between the two measurement methods however it does not quantify accuracy [33,34]. Accuracy between the measurement methods was quantified by additionally applying the root mean squared error (RMSE) analysis [35]. To further visualize the difference between the two-measurement methods Bland-Altman analysis was used [36]. Within the Bland-Altman analysis, the mean (m) and standard deviation (σ) of the differences between both measurement methods were computed. The values of m ± 2σ were also determined, as most of the differences (95% for the assumed Gaussian distribution) are expected to fall between these two limits. This procedure was previously used in Dadakova et al. [37]. The regression analysis, RMSE and Bland-Altman evaluations were carried out using the R statistical package (version 3.2.4, 2016, The R Foundation for Statistical Computing, Vienna, Austria). 

## 3. Results

### 3.1. Influence of Cable Length 

Cables of different lengths are used to connect the hyperthermia amplifier and the 12 dipole antenna pairs of each system, and consequently, the power lost on the cables differs among systems. Power losses on the cables at the five institutions are: 30.3%, 25.3%, 25.8%, 23.3%, and 26.5%, respectively for Institution 1 to 5. 

### 3.2. MRT vs. Thermistor Probe Measurements Evaluation 

#### 3.2.1. Centric Heating Target

**Figure 2:**
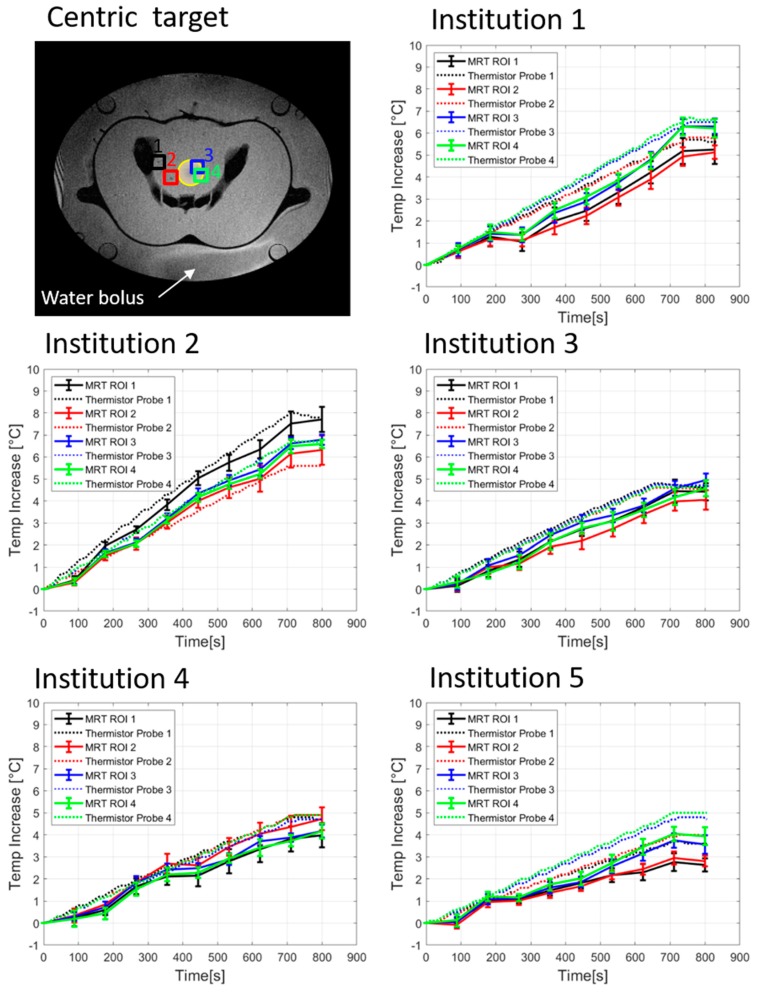
Temporal evolution of temperature increase determined by high resistance thermistor probes and by computed MRT for centric heating target (yellow circle). The position of probes and ROIs in the phantom are indicated by numbers and coloured squares in the MR image showing the cross-section of the phantom and the surrounding water bolus. Measurements were performed at five different institutions.

**Figure 3:**
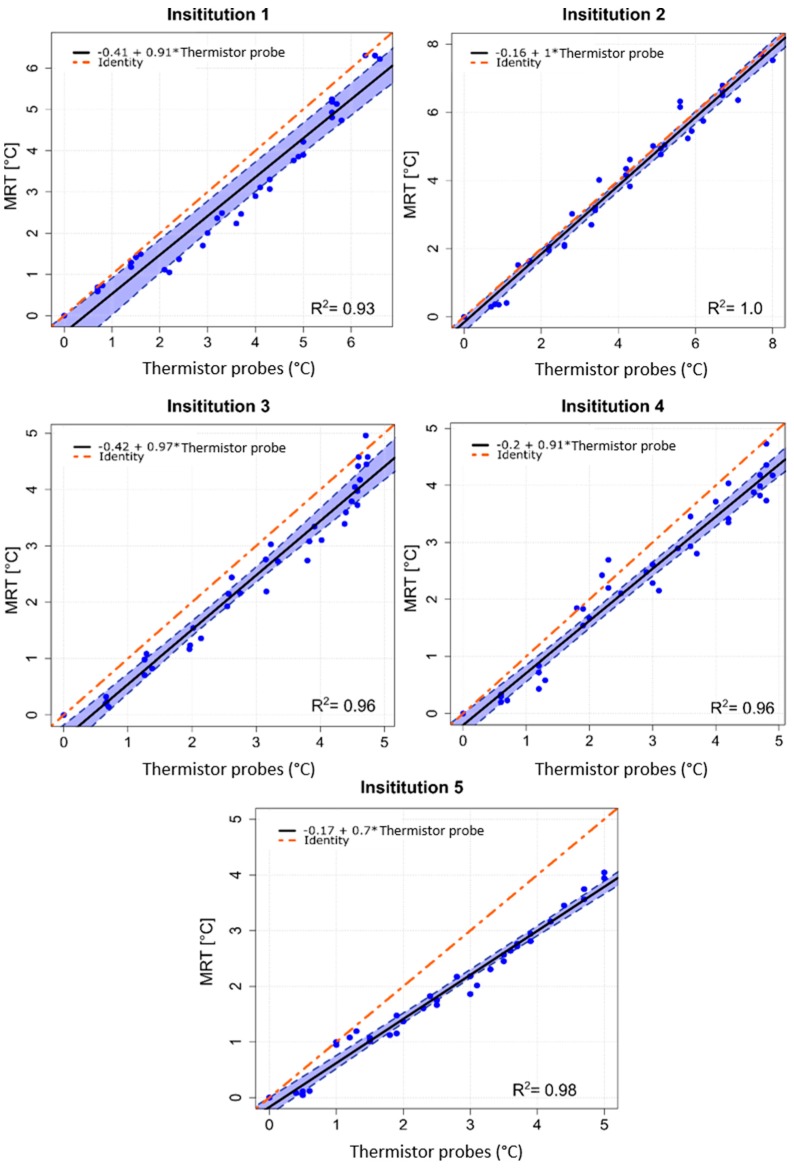
Scatter plots of MRT data as a function of high resistance thermistor probes measurements for centric heating target, measurements performed at the five institutions. The estimated linear regression equation (solid black line), the 95% confidence interval upper and lower limits (dashed blue lines) and the identity line of both methods (dot-dashed red line) are shown for all institutions. *R*^2^ coefficients are reported.

**Figure 4:**
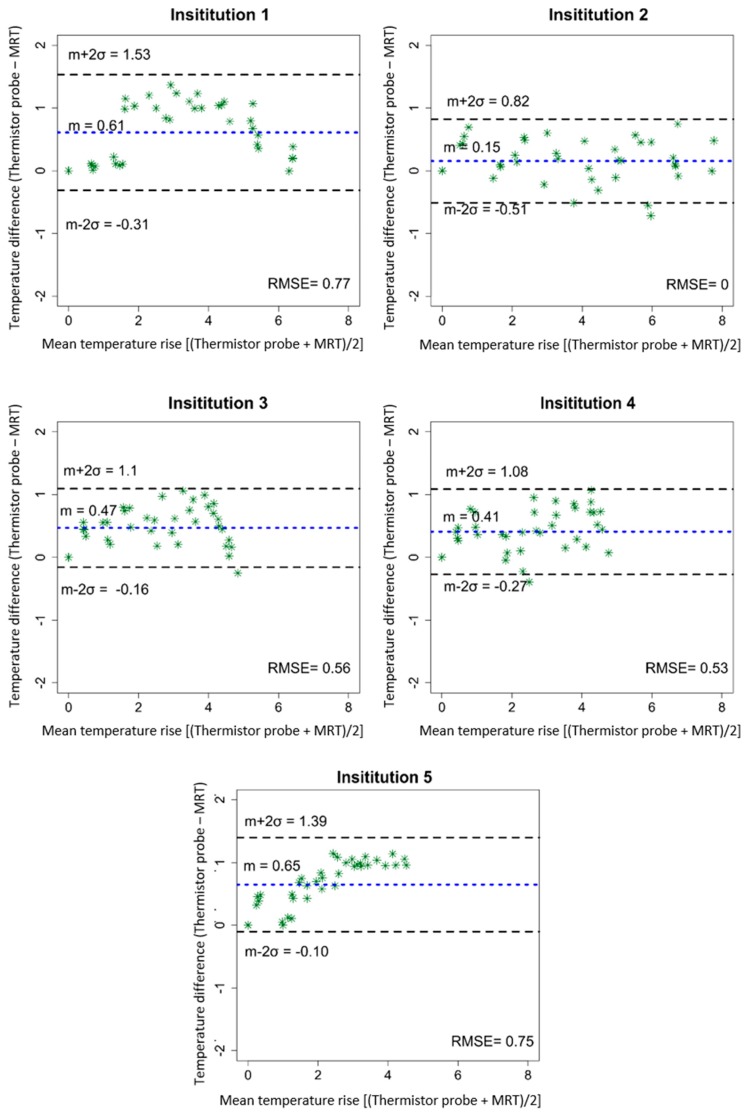
Bland-Altman quantitative analysis plots for centric heating target. The plots show the differences in the temperature change obtained by high resistance thermistor probes and MRT against the averages of the two measurements. The dotted blue line shows the mean difference, while the dashed black lines show the mean difference ± 2 × standard deviations. RMSE coefficients are reported.

The temporal evolution of temperature increase determined by thermistor probes and by computed MRT for a centric heating target is shown in Figure 2. The four evaluated locations are indicated in Figure 2 top left. Table 2 shows the temperature increases for all 4 thermistor probes and the corresponding average of MRT in the evaluated ROIs after 600 sec of heating. Minor differences in system responses can be seen at the 5 institutions, though the mean temperatures (thermistor probe and MRT) all agree within 1 °C. Institution 1 showed that the more centric locations 3 and 4 featured more temperature increase compared to the off-centric locations 1 and 2, which is in accordance with the intended centric target. The average temperature increases (±SD) at 600 sec were 5.0 ± 0.3 °C and 4.0 ± 0.3 °C for thermistor probes and MRT, respectively. Institution 2 showed higher temperature increase at location 1 compared with the other evaluated locations. The average temperature increase at 600 sec was 5.7 ± 0.7 °C for the thermistor probes and 5.4 ± 0.5 °C for the MRT. Considering Institution 3, the mean temperature increase was 4.3°C ± 0.1°C for the thermistor probes and 3.5 ± 0.2 °C for the MRT. Institution 4 featured the lowest power loss in the cables between amplifier and antenna elements, however temperature increase at this institution was not the highest compared to other institutions. The mean temperature increase at 600 sec was 4.0 ± 0.1 °C for the thermistor probes and 3.5 ± 0.3 °C for the MRT. The fifth institution featured an average value of the temperature increase at 600 sec of 3.7 ± 0.4 °C and 2.7 ± 0.4 °C for the thermistor probes and MRT, respectively. Scatter plots in Figure 3 show the MRT in comparison to thermistor probes measurements for centric heating target for the four-probe locations. For each institution a highly linear relation was determined. The mean *R*^2^ across the five institutions was found to be 0.97 ± 0.03 (0.93–1.0). As a qualitative note, results in Figure 3 enlighten that the computed MRT slightly underestimates the temperature measured by thermistor probes. The Bland–Altman plots for thermistor probes and MRT for centric heating target are shown in Figure 4. The overall mean (m) temperature difference (thermistor reading is higher than MRT) and standard deviation (σ) between both measurement methods are m = 0.46 ± 0.20 (0.15–0.65) °C and σ = −0.36 ± 0.06, respectively. The m − 2σ was found to be (−0.27 ± 0.16) °C and m + 2σ = (1.18 ± 0.28) °C. The RMSE across the five institutions were found to be 0.52 ± 0.31 (0–0.77) °C. 

#### 3.2.2. Eccentric Heating Target

**Figure 5:**
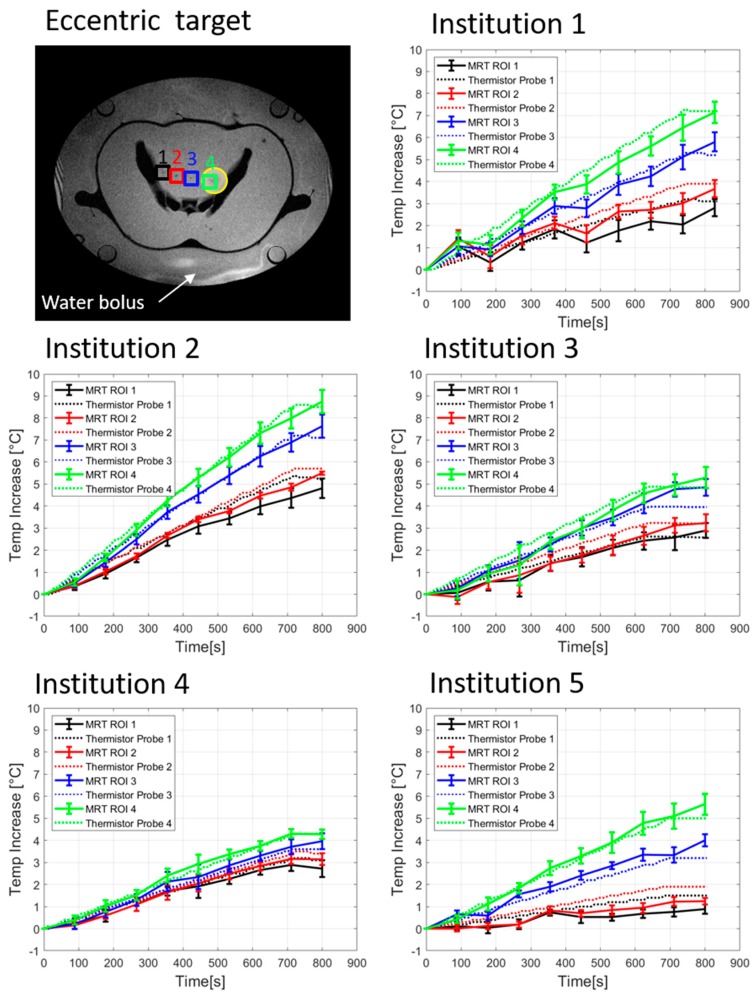
Temporal evolution of temperature increase determined by high resistance thermistor probes and by computed MRT for eccentric heating target (yellow circle). The position of probes and ROIs in the phantom are indicated by numbers and coloured squares in the MR image showing the cross-section of the phantom and the surrounding water bolus. Measurements were performed at five different institutions.

**Figure 6:**
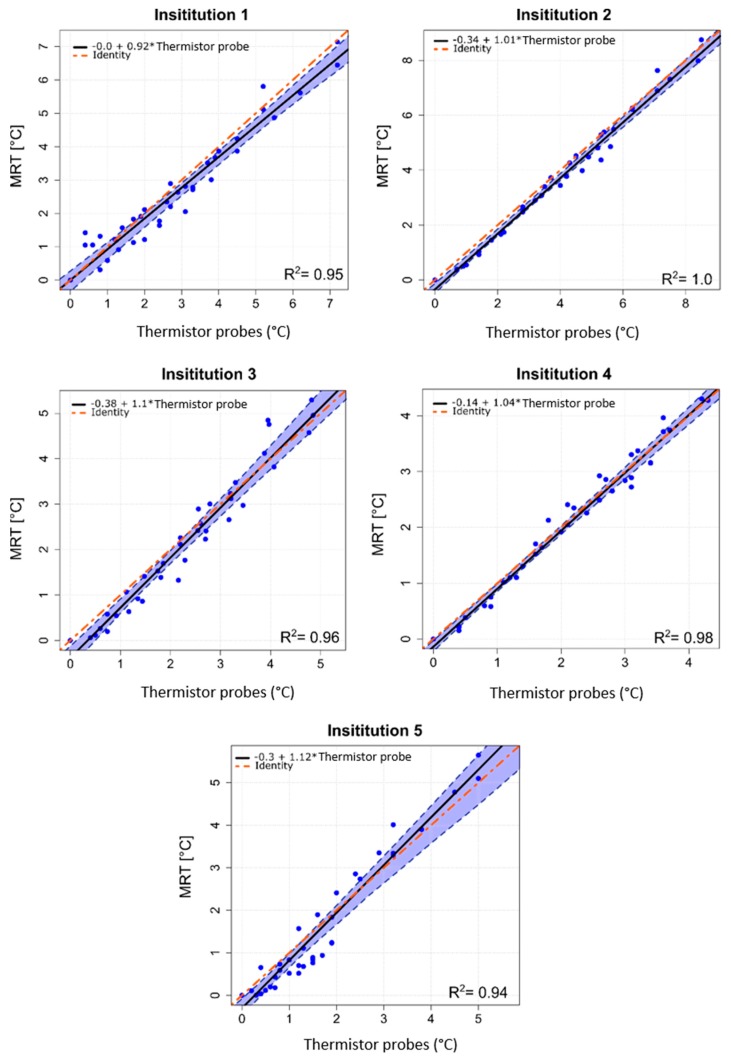
Scatter plots of MRT data as a function of high resistance thermistor probes measurements for eccentric heating target, measurements performed at the five institutions. The estimated linear regression equation (solid black line), the 95% confidence interval upper and lower limits (dashed blue lines) and the identity line of both methods (dot-dashed red line) are shown for all institutions. *R*^2^ coefficients are reported.

**Figure 7:**
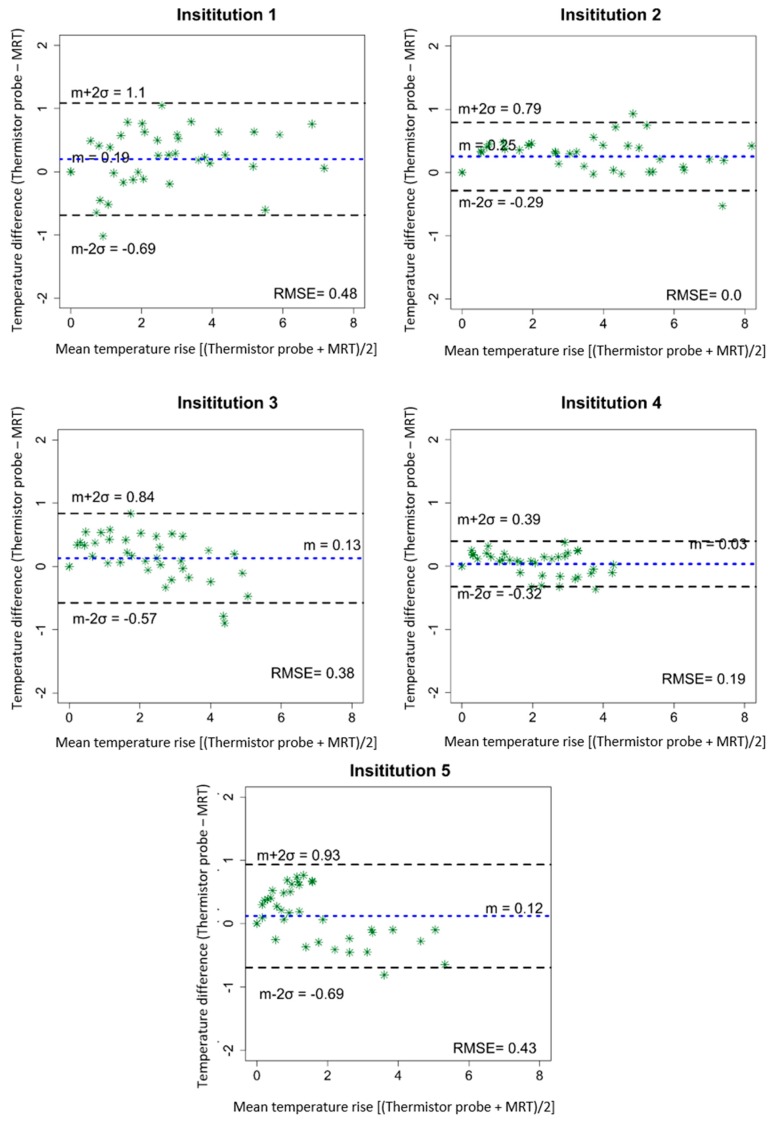
Bland-Altman quantitative analysis plots for eccentric heating target. The plots show the differences in the temperature change obtained by high resistance thermistor probes and MRT against the averages of the two measurements. The dotted blue line shows the mean difference, while the dashed black lines show the mean difference ± 2 × standard deviations. RMSE coefficients are reported.

Figure 5 illustrates the temperature increase of the thermistor probes as well as the computed MRT, for eccentric heating target. Table 3 shows the temperature increase for each thermistor probe and MRT at 600 sec of heating. For Institution 1, the mean temperature increase at 600 sec was 4.0 ± 1.2°C and 3.5 ± 1.3°C for the thermistor probes and MRT, respectively. Higher temperature increase could be seen in Institution 2 with an average temperature increase at 600 sec of 5.6 ± 1.1°C for the thermistor probes and 5.3 ± 1.3°C for the MRT. The acquired data of Institution 3 showed a mean temperature increase at 600 sec of 3.5 ± 0.8°C from the thermistor probes and 3.3 ± 0.9°C calculated from the MRT. Institution 4 featured a temperature increase at 600 sec of 3.0 ± 0.3°C for the thermistor probes and 3.0 ± 0.4°C for the MRT. Finally, the results of the quantitative evaluation of Institution 5 indicated the lowest average heating when compared to the other systems and preferential heating towards patient’s left side. The averaged values for the temperature increase at 600 sec were 2.5 ± 1.2°C and 2.3 ± 1.6°C for the thermistor probes and MRT, respectively. The scatter plots with regression lines (Figure 6) for eccentric heating target present a *R*^2^ value for all institutions of 0.97 ± 0.02 (0.94–1.0), additionally, good agreement between regression line and identity line was found. The Bland–Altman analysis for eccentric heating target (Figure 7) showed a mean (m) and standard deviation (σ) between both measurements equal to m = 0.13 ± 0.08 (0.03–0.25) °C, and σ = 0.32 ± 0.09, and consequently m − 2σ= (−0.52 ± 0.19) °C and m + 2σ= (0.81 ± 0.26) °C. The RMSE for all institutions was found to be 0.30 ± 0.20 (0–0.48) °C. 

## 4. Discussion

In this work, the performance of clinically used MR-guided deep hyperthermia applicators was for the first time systematically compared and evaluated in five European institutions in Germany and The Netherlands. For such comparison, a new dedicated phantom type with a realistic human-shape including implemented artificial bone structures was developed. Two phantoms of this type were transported to each of the five institutions where the same experimental procedure was applied for the QA measurements performed by the same principal investigator. Measurements were performed to evaluate the MRT vs. thermistor probes accuracy for centric and eccentric focus steering. 

The system efficiency in terms of power loss in the cables connecting the amplifier and antennas was determined and it is found to be 26.2 ± 2.3% across the five systems. The differences are explained by structural design conditions of the treatment rooms leading to variation in the cable lengths. However, this difference in power loss is not directly reflected in the temperature increase values. For example, Institution 1, which presents the highest power loss but not the lowest temperature increase. This indicates that other interrelated factors can be present such as differences in antenna matching, the coupling between antennas or phase induced errors at the antennas feed point [38,39].

The temporal evolution of thermistor probes and MRT change during the QA measurements (Figure 2 and Figure 5) of the five hyperthermia systems exhibit comparable heating performance but different maximum temperatures change for both heating targets. For centric heating target, the overall mean thermistor probe and MRT increase at 600 sec was 4.5 ± 0.7 °C and 3.8 ± 0.9 °C for the five institutions, respectively (Figure 2 and Table 2). This indicates that the temperature readings of all systems perform equally accurate. For eccentric target and evaluating Institutions 1, 2, 3, and 5, differences between the temperature increase at location 4 and location 1 (Figure 5 and Table 3, note that location 4 is near the eccentric focus whereas location 1 is farthest from the focus) were 2.8 ± 0.4 °C and 3.1 ± 0.7 °C, for thermistor probes measurements and MRT, respectively. This indicates good overall steering of heating target. If Institution 4 was also considered, differences between the temperature increase at location 4 and location 1 reduced their mean value and increased the SD, to 2.4 ± 0.8 °C for thermistor probes and 2.7 ± 1.0 °C for MRT. This indicates a different steering capability of the system at Institution 4 compared with the rest of the systems. However, the linear regression and Bland-Altman evaluation of Institution 4 indicates a very good correlation and agreement between thermistor probes and MRT measurements. 

The excellent correlation between MRT and invasive thermistor probes was quantified by the linear regression analysis with *R*^2^ of 0.97 ± 0.03 (0.93–1.0) for centric heating target and *R*^2^ of 0.97 ± 0.02 (0.94–1.0) for eccentric heating target (Figure 3 and Figure 6). Closer agreement between regression line and identity line was found for the measurements of eccentric heating as compared to the central heating. This might be attributed to the use of one phantom for all central heating measurements and another phantom for all eccentric heating measurements. A positioning error of the thermistor probes could have led to this correlation difference. Additionally, the analysis also illustrates a potential setup error at Institution 5 for centric heating target, in which some of the thermistor probes could have been slightly miss-positioned with respect to the MRT axial central plane. This effect was not observed for the measurements for eccentric target. 

The Bland-Altman analysis (Figure 4 and Figure 7) showed an overall mean difference between thermistor probes measurements and MRT of 0.46 ± 0.20 (0.15–0.65) °C and 0.13 ± 0.08 (0.03–0.25) °C for centric and eccentric heating targets, respectively. The RMSE was found to be 0.52 ± 0.31 (0–0.77) °C for centric target and 0.30 ± 0.20 (0–0.48) °C for eccentric target. As described above, the larger overall mean difference between thermistor probes and MRT for centric target in comparison to eccentric target can be attributed to the use of two different phantoms. Even that the phantoms were constructed in an identical way, the independent alignment of the probes under CT-guidance can lead to sub-centimetre errors in the identification of the probe positions. Nevertheless, the differences between thermistor probes and MRT found, are in agreement with previous publications in which a temperature difference of 1 °C was considered an acceptable clinical limit for the application of hyperthermia [40]. The difference that we found in our study between both measuring methods is also within the agreement with more recent works evaluating MRT with invasive temperature probes for deep hyperthermia [11,26,27,28,37] and also for local hyperthermia using a head and neck applicator [22,25]. Additionally, in previous publications, the evaluation was performed in only one hybrid device at one institution, while our study evaluates the performance of five hybrid devices. 

The performed measurements show comparable results between the systems of the five evaluated institutions. The obtained differences between measured thermistor probes and MRT are within the previously reported values, below 1 °C, and for all the evaluated institutions. The differences obtained between centric and eccentric heating targets indicate that a more accurate thermistor probe positioning procedure is needed. Additionally, multiple measurements for each phantom setup and institution would be required to evaluate the reproducibility of the results. Furthermore, it is important to mention that three different MR-system types from various vendors (Siemens, Philips, GE) are installed within the five institutions. The three types of MR-systems feature differences in terms of magnet quality, technical system performances and sequence parameters of the thermometry protocol (Table 1), which together may cause a slight variation in the MRT accuracy among the five institutions. This study indicates that the Universal Applicator performs equally as the other systems using a Sigma Eye applicator with this setup and a limited number of experiments. The results of this study will help to develop QA procedures and deliver more uniform treatment quality among institutions in multi-centre trials. Together this can reflect in improved integration of hyperthermia into the patient treatment process and thus contribute to achieving higher acceptance in the radiotherapy community.

## 5. Conclusions

This paper reports the results of the first international multi-institution QA evaluation of hyperthermia systems in terms of MRT accuracy. This evaluation has been performed identically in all five institutions equipped with MR-guided hyperthermia systems for the treatment of deep-seated tumours. An anthropomorphic phantom with human representative dimensions has been developed, and it has shown to be a valuable tool for QA measurements of the systems that can better reflect the clinical situation. Good agreement between thermistor probe measurements and MRT was found within and between institutions for both central and eccentrically located targets. An excellent linear correlation between thermistor probes measurements and MRT was obtained. This study demonstrates the feasibility of inter-institutional QA-related to MR-thermometry and provides a basis to define standards for the delivery of treatments in MR-guided multi-institutional studies and to elucidate thermal dose-effect relations.

## Figures and Tables

**Table 1:** MR sequences parameters as used during the clinical routine and during the performed experiments at the five institutions, for High Resolution scans and Magnetic Resonance Thermometry (MRT) scans.

Institution	Scan Type	TR (ms)	TE1 (ms)	TE2 (ms)	FA (deg)	Acquisition Matrix	Reconstruction Matrix	Scan Time (sec)
1	High Resolution	120	4.8	9.60	70	256/256	256/256	136
	MRT	620	4.8	19.1	40	128/128	256/256	83
2 4 5	High Resolution	120	4.76	9.53	70	256/256	256/256	124
	MRT	600	4.76	19.10	50	128/128	128/128	78
3	High Resolution	120	4.60	9.21	70	252/250	256/256	151
	MRT	600	4.60	18.42	50	128/128	256/256	79

**Table 2:** Temperature increase at 600 sec for centric heating target measured with thermistor probes and computed Magnetic Resonance Thermometry (MRT).

Institution	Temperature Measurement	Mean Temperature Increase [ΔT (°C)] at Locations 1 to 4				Mean Temperature Increase± SD (°C)
		ΔT Location 1	ΔT Location 2	ΔT Location 3	ΔT Location 4	
1	Thermistor probe	4.7	4.6	5.2	5.3	5.0 ± 0.3
	MRT	3.8	3.5	4.3	4.3	4.0 ± 0.3
2	Thermistor probe	6.8	4.7	5.6	5.6	5.7 ± 0.7
	MRT	6.2	4.9	5.3	5.1	5.4 ± 0.5
3	Thermistor probe	4.4	4.2	4.4	4.3	4.3 ± 0.1
	MRT	3.6	3.2	3.7	3.5	3.5 ± 0.2
4	Thermistor probe	4.1	4.1	3.9	4.0	4.0 ± 0.1
	MRT	3.2	3.9	3.5	3.3	3.5 ± 0.3
5	Thermistor probe	3.2	3.4	4.0	4.2	3.7 ± 0.4
	MRT	2.3	2.4	3.0	3.3	2.7 ± 0.4

**Table 3:** Temperature increases at 600 sec for eccentric heating target measured with thermistor probes and computed Magnetic Resonance Thermometry (MRT).

Institution	Temp. Measurem.	Mean Temperature Increase [ΔT (°C)] at Locations 1 to 4				Mean Temp. Increase ± SD (°C)	d(ΔT Location 4- ΔT Location 1) (°C)
		ΔT Location 1	ΔT Location 2	ΔT Location 3	ΔT Location 4		
1	Thermistor probe	2.6	3.2	4.3	5.9	4.0 ± 1.2	3.3
	MRT	2.0	2.7	4.1	5.3	3.5 ± 1.3	3.3
2	Thermistor probe	4.5	4.7	6.1	7.2	5.6 ± 1.1	2.7
	MRT	3.8	4.3	6.0	7.0	5.3 ± 1.3	3.2
3	Thermistor probe	2.5	3.0	3.7	4.6	3.5 ± 0.8	2.1
	MRT	2.3	2.5	3.9	4.4	3.3 ± 0.9	2.0
4	Thermistor probe	2.7	2.9	3.0	3.6	3.0 ± 0.3	0.9
	MRT	2.6	2.8	3.2	3.7	3.0 ± 0.4	1.1
5	Thermistor probe	1.3	1.7	2.8	4.3	2.5 ± 1.2	3.0
	MRT	0.6	0.9	3.2	4.6	2.3 ± 1.6	3.9
